# The Crosstalk Between *Mycobacterium abscessus* and Immune Cells: Exploring Novel Interaction Modalities

**DOI:** 10.3390/cells14221829

**Published:** 2025-11-20

**Authors:** Ilse Mendoza-Trujillo, Patricia Diez-Echave, Chiara Tontini, Silvia Bulfone-Paus

**Affiliations:** 1 Lydia Becker Institute of Immunology and Inflammation, School of Biological Sciences, Faculty of Biology, Medicine and Health, University of Manchester, Manchester M13 9NT, UKchiara.tontini@manchester.ac.uk (C.T.); 2 Allergy Research Group, Instituto de Investigación Biomédica de Málaga y Plataforma en Nanomedicina-IBIMA Plataforma BIONAND, 29590 Málaga, Spain

**Keywords:** *Mycobacterium abscessus*, bacterial infections, immune cells, host–pathogen interactions, non-tuberculous mycobacteria (NTM)

## Abstract

*Mycobacterium abscessus* (*Mab*) is a rapidly growing, non-tuberculous mycobacterium and opportunistic pathogen that causes lung and skin infections in immunocompromised individuals. In recent years, *Mab* has gained attention due to its resistance to multiple antibiotics and its ability to evade the immune response by transitioning into different morphotypes. Macrophages and neutrophils play key roles during the acute phase of infection and granuloma formation, utilising clearance mechanisms that affect the smooth and rough morphotypes differently. Despite considerable research, the inflammatory response elicited by *Mab* and its impact on disease outcomes remain not well understood. This perspective examines the interactions between Mab and immune cells, proposing potential receptors that may mediate *Mab*-driven immune communication. By drawing insights from immune evasion and signalling strategies employed by other mycobacterial species, it aims to deepen our understanding of *Mab* pathogenicity and to outline innovative approaches for infection control.

## 1. Introduction

Non-tuberculous mycobacteria (NTM) are bacteria commonly found in the environment, particularly in soil, natural water sources, and biofilms. Although individuals are frequently exposed to NTM, these bacteria are rarely able to cause diseases due to the generally low pathogenicity of most NTM species [1]. NTM include, among others, *Mycobacterium avium complex* (*M. avium*, *M. intracellulare* and *M. chimaera*). *M. kansasii*, *M. marinum*, *M. ulcerans* and *M. smegmatis*.

*M. abscessus* (*Mab*), an opportunistic, rapidly growing mycobacterium species, is considered the second most prevalent and most pathogenic among NTM, accounting for 3–13% of isolates in pulmonary disease [2], and the leading causative agent of extrapulmonary infections [3,4,5]. In recent years, there has been a notable increase in infections caused by *Mab*, especially among paediatric patients. This could be the result of increased awareness and improved diagnostics [6], but also raises significant concerns over its increased transmissibility and pathogenicity [7]. Although the *Mab* transmission dynamics are not entirely understood, contaminated fomites and inhalation of aerosolized bacilli from showerheads have been proposed as likely environmental causes [8]. However, increasing evidence over the past few decades suggests that direct person-to-person transmission is becoming more common globally [9].

A combination of immune and predisposing medical conditions influences susceptibility to infection by *Mab*. Disseminated *Mab* infections have been reported in immunocompromised individuals, with innate, acquired, or medication-induced defects in the IL-12-IFN-γ and TNF-α pathways [10,11,12]. Furthermore, *Mab* tends to colonize the airways in chronic lung conditions such as cystic fibrosis (CF), chronic obstructive pulmonary disease, bronchiectasis, or prior history of tuberculosis [13]. Persistent lung infections are likely to be facilitated by anatomical disruption [14], local impairments in the production of innate anti-microbial enzymes/peptides, like α1-antitrypsin and β-defensins, as well as reduced cytokine release, particularly defective IL-6 and IL-17 responses, alongside increased levels of circulating anti-IL-1Rα receptor antagonist [12,15,16]. While healthy subjects are typically resistant to *Mab* infection, there are multiple reports of skin and soft tissue infections stemming from traumatic injury or cosmetic/surgical procedures with contaminated equipment [17,18].

Once the infection is established, *Mab* is managed with a triple antibiotic combination, following a similar approach to other mycobacterial infections, for a minimum of 12 months once cultures become negative [19]. While current practice parameters suggest choosing the initial antibiotic combination based on bacterial resistance tests [19], there is no consensus on the most effective treatment combination to date [20].

The main reason why *Mab* infection is particularly challenging to treat is due to its intrinsic resistance to several classes of antibiotics, including aminoglycosides, beta-lactams, rifampicin, macrolides, tetracycline, and doxycycline [21]. A 2019 meta-analysis by Kwak et al. reported therapeutic success rates ranging between 33–69% [22]. Additionally, a systematic review indicated an increased five-year mortality in the presence of comorbidities, male sex, and fibro-cavitary disease, with the highest mortality observed in patients with persistent NTM infections [23].

Given the rising prevalence and associated morbidity and mortality, there is an urgent need for new preventive and therapeutic strategies against *Mab*, including both direct bactericidal agents and immunomodulatory strategies to increase bacterial recognition and clearance. However, information on *Mab* pathogenesis from basic research and clinical observations is still limited.

Considering the paucity of knowledge on *Mab*, we performed a thorough PubMed and Web of Science review of currently available evidence from *Mab* and other mycobacterial species.

In this perspective, we first discuss the known mechanisms by which host immune cells interact with *Mab* and how these interactions initiate downstream immune responses. However, current knowledge of *Mab*–host cell receptor interactions remains limited. To address this gap, we examine host cell receptors that mediate interactions between immune cells and other NTM, assessing their roles in immune responses and their potential relevance to *Mab* infection. By drawing parallels between mycobacteria interactions across different NTM species, we propose possible mechanisms contributing to *Mab* pathogenesis and immune evasion. We also highlight promising targets for future therapeutic development.

## 2. *Mycobacterium abscessus*: Subtypes and Pathogenesis

A particularly interesting feature of *Mab* is the composition of its cell envelope. The distinct lipids on the *Mab* surface, drug efflux pumps, and secretion systems are vital in modulating bacterial infection dynamics, virulence, and pathogenesis.

*Mab* exhibits different morphotypes, transitioning from a smooth to a rough form due to the loss of major surface glycopeptidolipids (GPLs) [24,25,26]. These surface molecules are associated with various characteristics, such as aggregation, sliding motility, and biofilm formation [27,28]. In contrast, the rough variants lack GPLs, do not form biofilms, and are non-motile but form cords. This morphotype is associated with more severe clinical manifestations and an enhanced ‘hyper-proinflammatory’ response, a phenomenon observed in different models, including clinical as well as in vitro and in vivo studies [29,30,31].

Evidence suggests that the smooth morphotype initiates infection by entering the host and colonizing tissue, eventually transitioning to the rough form as the infection becomes chronic [32,33]. This hypothesis is supported by murine model studies demonstrating the spontaneous emergence of rough mutants from infections initially caused by *Mab* smooth variants [34,35,36,37]. Moreover, clinical evidence indicates that most isolates from individuals with chronic lung disease exhibit the rough phenotype. In contrast, isolates from wounds contaminated by environmental sources predominantly display the smooth phenotype [26].

Although mutations that drive the transition from smooth to rough are known (*MmpL4* mutation, or mutation or silencing of *mps1*, *mps2* and *gap* genes) [38], the mechanisms driving this morphotype conversion remain unclear. In vivo studies suggest that interactions between the bacterium and the host immune system during infection may facilitate this transition [30,39]. In parallel, in vitro studies indicate that antibiotic pressure [40] and oxidative stress [41] can also induce the conversion. Both variants can co-exist in the patient and evolve differently in response to the host’s anti-microbial response [42,43].

**Figure 1:**
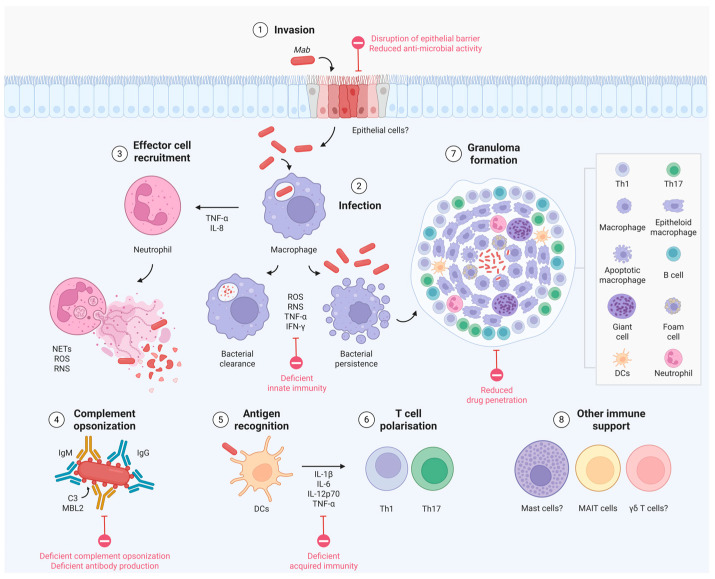
Immune mechanisms involved in *Mycobacterium abscessus* infection. Schematic depiction of current knowledge of the immune events following invasion and infection induced by *Mycobacterium abscessus* (*Mab*) and other nontuberculous mycobacteria (NTM). Facilitating host factors are highlighted in red. Question marks (?) indicate cells with possible involvement based on evidence from other NTM. Mycobacteria colonise epithelial cell surfaces and disrupt the epithelial cell barrier [45,46,47,48,49]. Upon entry (**1**), *Mab* first encounters innate immune cells, like macrophages, that recognise and phagocyte mycobacteria [44]. Depending on the host’s immune status, macrophages can clear the infection via intracellular killing mechanisms, or fail to mount an effective antibacterial response, thus letting *Mab* survive and proliferate intracellularly [44] (**2**). Infected macrophages participate in the recruitment of granulocytic effector cells, like neutrophils [50], through the release of cytokines and chemokines (**3**), which participate in *Mab* clearance via intra- and extracellular killing mechanisms [37], alongside complement opsonization [30] (**4**), facilitating the clearance and recognition of mycobacteria by antigen-presenting cells (**5**). Antigen-presenting cells, like dendritic cells, process mycobacterial antigens and drive T cell polarisation towards NTM-specific proinflammatory responses [44,51,52] (**6**). This concerted immune response results in the formation of granulomas (**7**), which isolate infected cells and mycobacteria within layers of activated cells of the innate and adaptive immune system [50,53]. Other immune actors (**8**), like mast cells [54], γδ T cells [55] and MAIT cells [56], could be involved in *Mab*/NTM infection by mounting anti-mycobacterial responses and/or influencing the granuloma microenvironment. Figure created using Biorender.com. Abbreviations: C3, complement factor 3; DCs, dendritic cells; IFN-γ, interferon gamma; Ig, immunoglobulin; IL, interleukin; MBL2, mannose binding lectin 2; NET, neutrophil extracellular traps; ROS, reactive oxygen species; RNS, reactive nitrogen species; Th, T helper cells; TNF-α, tumour necrosis factor alpha. References: *Mab* [30,37,44,49,50,51,52,53], other NTM [45,46,47,48,54,55,56].

As with other mycobacteria, an early innate immune response involves the recognition and engulfment of bacteria by phagocytic cells, macrophage activation, and the release of proinflammatory cytokines to promote intracellular killing. This is followed by the formation of granuloma and, in unresolved cases, the establishment of chronic infection [44] (Figure 1).

The initial step in *Mab* infection involves the recognition of antigens through pathogen recognition receptors (PRRs) on the immune cells’ surface (macrophages and dendritic cells, DCs). Both morphotypes can be phagocytosed by macrophages that employ mechanisms like reactive oxygen species (ROS) and reactive nitrogen species for intracellular killing [44]. Neutrophils also contribute to host defence by being recruited to the site of infection after macrophages release cytokines [37].

As the infection progresses, an increasing number of macrophages and other immune cells gather at the site, forming structures known as granulomas. These immunological structures, considered the hallmark of mycobacterial infections, consist of clusters of host immune cell subsets that organise to contain the bacteria and limit their spread [1,53]. In zebrafish models, *Mab* granulomas are composed primarily of neutrophils and macrophages, and their formation heavily relies on TNF signalling and IL-8–mediated neutrophil recruitment [50]. The contribution of adaptive immune cells, particularly T cells, has also been highlighted. In patients infected with *Mab*, the T helper 1 (Th1)/T helper 2 (Th2) balance is reduced while T helper 17 (Th17) cytokines are elevated compared to healthy controls. This observation suggests that Th1 polarization may promote protective immune responses during infection [44].

Granuloma formation is primarily observed in cutaneous lesions, characterized by a typical pattern of nodular inflammation. These lesions often feature central abscesses surrounded by epithelioid and multinucleated giant cells [57]. In immunosuppressed patients, the inflammatory infiltrate can spread diffusely into the deep dermis and subcutaneous tissue, and granulomas are most likely to present in a suppurative form. By contrast, immunocompetent patients tend to exhibit granulomas that resemble those seen in sarcoidosis [58].

Clinically, cutaneous manifestations include localized erythema, plaques, purplish-red nodules, ulcers, and painless abscesses [3,59]. In pulmonary infections in immunocompetent patients, cavitary and consolidative lesions are often found, particularly in the upper lobes. Chest CT scans show widespread branching nodular opacities or a tree-in-bud pattern, bronchiectasis, and small nodules [60].

## 3. Host Immune Response: Pathways and Modalities

Following exposure, *Mab* is recognized by pattern recognition receptors (PRRs) such as Toll-like receptor 2 (TLR2), Dectin-1, and scavenger receptors that play a pivotal role in recognizing bacterial cell wall components and triggering innate immune responses. PRRs facilitate interactions between neutrophils, macrophages, and dendritic cells with *Mab*, activating signalling pathways that lead to the production of pro-inflammatory cytokines, including TNF-α and IL-1β [61].

Several studies have established the critical role of TLR2 in *Mab* infections [62,63,64]. Specifically, the rough morphotype has proven to be a more potent activator of this signalling pathway than its smooth counterpart [65]. The absence of GPLs in the cell membrane in the rough form leads to an increased exposure of other lipoproteins on the bacterial surface that promotes an overactivation of TLR2-dependent mechanisms in macrophages, such as TNF-α production and NF-κB activation [63]. Moreover, *Tlr2^−/−^* mice infected with the rough morphotype exhibit impaired early Th1-type adaptive immunity, characterized by reduced production of IFN-γ, TNF-α, and IL-12p70, diminished immune cell recruitment to the infection site, and decreased activation of dendritic cells and T cells, ultimately resulting in poor survival rates [64].

Notably, co-immunoprecipitation and confocal microscopy have shown that Dectin-1 physically interacts with and co-localizes with TLR2 in macrophages during *Mab* infection. This suggests their synergistic role in the immune response elicited by *Mab* [61]. However, studies in Dectin-1/*Clec7a*-deficient mice have demonstrated that these mice do not exhibit impaired immune responses to *Mab*, indicating that multiple mechanisms are in place to regulate host–mycobacterial interactions [66].

Other receptors involved in host defence have also been investigated, such as NOD2, particularly in the context of the rough morphotype [67]. A *Nod2*-deficient murine model displayed impaired bacterial clearance and more severe lung pathology following *Mab* infection. This phenotype was associated with reduced production of proinflammatory cytokines, including IL-6, TNF-α, and IL-1β, as well as diminished nitric oxide (NO) synthesis, all of which are critical for effective infection control [67]. Furthermore, these findings underscore the impact of *Mab*’s outer membrane and its distinct rough/smooth subtypes in modulating the engagement of different innate immune pathways across various immune cell populations.

Macrophages and neutrophils play a crucial role in forming granuloma-like structures in response to *Mab* infection. The recruitment of neutrophils to the infection site is primarily mediated by cytokines such as IL-8 and TNF-α. Notably, inhibiting TNF receptor 1 (TNFR1) expression not only reduces neutrophil recruitment but also leads to the formation of disorganized granuloma-like structures, characterized by loose and unstructured formations [68]. This disruption of the TNF-α signalling pathway impairs IL-8-dependent neutrophil recruitment, resulting in abnormal granuloma formation and significant mycobacterial accumulation [50]. Furthermore, infection with *Mab* has been shown to worsen during immunosuppressive therapy involving TNF-α inhibitors, further emphasizing the critical role of TNF-α in regulating the immune response to this pathogen [69].

Beyond phagocytosis, neutrophils also exert extracellular mycobactericidal activity by forming neutrophil extracellular traps (NETs), which appear to be effective in controlling both *Mab* morphotypes [70]. In addition to NET formation, another important mechanism in bacterial clearance involves complement opsonization, which enhances bacterial killing, although rough isolates are less susceptible to complement-mediated clearance. Complement components C3 and mannose-binding lectin 2 (MBL2) protein bind to the morphotypes in distinct patterns, with more MBL2 protein binding to rough strains [30]. Killing is C3-dependent, but not through traditional complement pathways. Instead, natural IgG and IgM mediate killing of smooth morphotypes, while IgG alone is required for rough ones. Both morphotypes are recognized by complement receptor 3 in a carbohydrate- and calcium-dependent manner. These findings suggest a noncanonical C3 activation pathway for neutrophil-mediated *Mab* clearance, further linking morphotype adaptation to complement activation [30].

In addition to these cellular interactions, macrophage activation and polarization during *Mab* infection are also influenced by proinflammatory mediators, including proteins, lipids, and nucleic acids present in exosomes secreted by human bronchial epithelial cells. These microvesicles have been shown to upregulate the expression of the M2 macrophage marker gene *ARG1* (arginase 1), while simultaneously downregulating M1-associated markers like IL-1β and IL-6 in human macrophages. Since M2 macrophages create an environment conducive to mycobacterial survival and replication, their increased activation and expansion could contribute to the host’s resistance to clearing smooth *Mab* infection [71].

Finally, dendritic cells, when stimulated via TLR4 by *Mab* antigens, such as the identified D-alanyl-D-alanine dipeptidase (MAB1843), undergo maturation and produce key pro-inflammatory cytokines, including IL-12p70, IL-1β, IL-6, and TNF-α. These cytokines contribute to T cell polarization and promote IFN-γ production by Th1 cells, emphasizing the central role of dendritic cells in initiating a Th1-type immune response and directing the host’s anti-mycobacterial activities [51,52].

## 4. The Role of Surface Lipids in *Mycobacterium abscessus* Virulence

As previously mentioned, the main difference between the two *Mab* morphotypes depends on their membrane composition [32]. The smooth morphotype variant of *Mab* has a GPL layer that masks TLR agonists, such as phosphatidylinositol mannosides and lipoproteins. This masking prevents pathogen recognition, thus helping *Mab* evade the host organism’s innate immune response [38].

Research on surface lipids has primarily focused on their role in immune evasion, particularly concerning cell death. For example, the smooth morphotype recruits the phagosomal marker Rab5, preventing phagolysosome acidification in macrophages. As a result, pH-dependent proteases and lipases remain inactive and cannot contribute to bacterial killing. In contrast, the surface lipids exposed in the rough morphotype trigger early apoptotic events but inhibit the fusion of autophagosomes with lysosomes, thereby facilitating bacterial persistence within host cells and ultimately promoting pathogen dissemination [72]. Moreover, the rough strain lipid composition also contributes to the production of mitochondrial ROS (mtROS), which can lead to the release of IL-1β by NLR family pyrin domain containing 3 (NLRP3) inflammasome activation and cGAS-STING-dependent type I IFN responses [73].

Despite this, most in vitro studies use the smooth variant as the reference standard, even though some isolates from patients with active disease exhibit a rough morphotype due to prolonged interaction with the host environment [42,74]. It is also important to note that both morphotypes often coexist, likely activating parallel pathways simultaneously contributing to persistence. Therefore, approaches that analyse both strains together are crucial to better understanding infection dynamics and developing more effective eradication strategies.

## 5. What Can We Learn from Other Nontuberculous Mycobacteria to Better Understand *Mycobacterium abscessus* Pathogenesis?

Although our understanding of the host immune response to *Mab* is largely based on canonical pathways, NTM display unique strategies for immune evasion, persistence, and disease progression, suggesting they may also activate alternative host responses. Exploring additional signalling pathways involved in NTM pathogenesis could reveal novel mechanisms driving *Mab* pathogenesis and identify new candidates for therapeutic targeting. This section first mentions receptors involved in *Mab* infection that remain poorly investigated. We then describe novel receptors characterized in other NTM infections, which have not yet been explored for *Mab*, but may point to promising candidate receptors for future research (Table 1).

Until now, TLR2 and Dectin-1 have been identified as receptors involved in the recognition of *Mab*. However, other receptors remain poorly characterized despite their potential roles in modulating immune functions. Several previously unexplored receptors have been recently proposed as key contributors to the host immune response against *Mab*. For instance, a genome-wide screen identified *CD46*, *ITGB2* (CD18), and *M6PR* (mannose-6-phosphate receptor) as relevant mediators during *Mab* internalization in human macrophages [75]. Additionally, CD81, a member of the tetraspanin family, proved essential for *Mab* uptake in an in vitro macrophage infection model [76].

Evidence supporting the role of complement receptors CR3 (CD11b/CD18) and CR4 (CD11c/CD18) in mediating NTM uptake is limited compared to that for tuberculous mycobacteria [77,78]. However, one study demonstrated that blocking the CR3 receptor markedly inhibited non-opsonic phagocytosis of *M. kansasii* by human neutrophils [79]. Similar results were reported in bovine monocytes infected with *M. avium* subspecies. *paratuberculosis* (MAP) where blocking CD18 resulted in a decreased uptake. In the same study, inhibition of CD14 or mannose receptors also impaired MAP uptake, suggesting that multiple receptor pathways cooperate in NTM internalization [80].

Studies on other NTM have also identified several host receptors that remain unexplored in *Mab* infections, such as intracellular TLRs. In studies involving *TLR9^−/−^* mice infected with formalin-killed *M. avium*, a decrease in the number of macrophages and lymphocytes in granuloma structures was observed compared to wild-type mice. However, the role of TLR9 in cytokine modulation remained unclear [81]. This is particularly noteworthy because TLR9 recognizes unmethylated CpG DNA motifs found in microbial DNA or endogenous DNA released from damaged host cells [82], likely present in mycobacterial lesions. These observations highlight the influence of the host environment and suggest the possibility that additional bacterial components may engage similar receptors, triggering alternative signalling pathways.

**Table 1:** Receptors implicated in NTM immune responses that may constitute potential targets in *Mab* research.

Receptor Protein	Receptor Family	Type of Study	Mycobacterial Species	Immune Response Involved	Ref.
**TLR9**	Toll-like receptor	In vivo	*M. avium*	Recruitment of macrophages and lymphocytes to the granuloma	[81]
**CR3** **(CD11b/CD18)**	Complement receptor	In vitro	*M. avium*, *M. kansasii*	Phagocytosis	[79,80]
**Mannose** **receptor**	C-type lectin (CLEC)	In vitro	*M. kansasii*	Phagocytosis	[80]
**CD14**	Glycosylphosphatidylinositol (GPI)-anchored receptor	In vitro	*M. kansasii*	Phagocytosis	[80]

## 6. Exploring the Contribution of Immune Resident Cells

**Figure 2:**
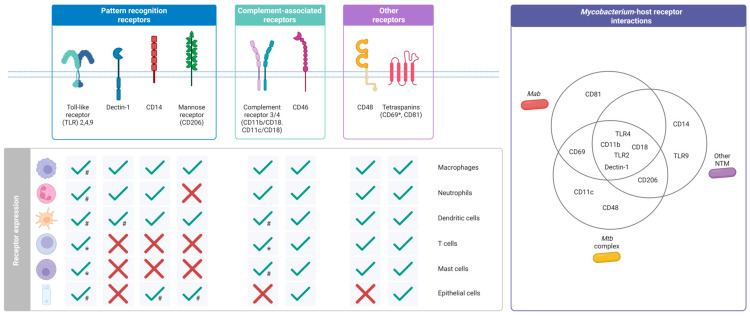
Proposed receptors involved in *Mycobacterium abscessus* pathogenesis. Schematic representation of receptors known to interact with *Mycobacterium abscessus* (*Mab*), other nontuberculous mycobacteria (NTM) or *Mycobacterium tuberculosis* (*Mtb*) complex and their relative expression by immune and non-immune cells. Asterisk symbol (*) indicates receptor expression observed during cell activation, while hash (#) receptor expression restricted to specific cell subsets/selected receptors. Receptor expression information by different cell types is based on the human protein atlas (https://www.proteinatlas.org/, last accessed 31 October 2025 [108]) and for mast cells on [109]. Activation-dependent receptor expression by mast cells based on [93] and T cells on [110,111]. Mycobacteria engage with different host immune receptors, particularly pattern recognition receptors, which include Toll-like receptors, Dectin-1, and mannose receptor CD206 [61,62,63,80], expressed by both immune and non-immune cell types. Different cell subsets and/or specific activating conditions can influence the expression of TLR receptors (e.g., TLR9 expression is confined intracellularly and exclusively observed in dendritic cells; mast cells and T cells might upregulate TLR2 and 4 following stimulation; CD14 can be expressed by epithelial cells in specific anatomic sites, like bladder and intestines) [93,110,112,113]. Complement receptors (CR) are formed by the aggregation of the surface integrin αV/CD11b and integrin αX/CD11c with integrin β2/CD18, to form CR3 and CR4, respectively, which was shown to interact with different mycobacterial species [77,78,79,80]. CR3/4 expression is predominantly observed in myeloid cells and some T cell subsets following activation (e.g., CR3 by CD8 T cells [111]). Some myeloid cells, like mast cells, might lose the expression of specific integrins (i.e., CD18) during maturation in tissues [114]. Conversely, the complement regulatory protein CD46, CD48 and the tetraspanin CD81 [75,76,90] are ubiquitously expressed by immune cells, while epithelial cells do not usually express CD48. Based on available literature [79,80,81], there is extensive overlap in the recognition of *Mab*, NTM and *Mtb* complex mycobacteria via TLR2/4, CR3 and Dectin-1, while evidence of CR4 and CD48 engagement is restricted to the *Mtb* complex, CD14 and TLR9 to NTM, and CD81 to *Mab*.

Macrophages and neutrophils have been extensively characterized as key players in *Mab*/NTM recognition and clearance. However, considerably less is known about the contribution of specific immune resident cells to host defence in *Mab* and other NTM infections due to the paucity of studies. Nevertheless, evidence from species belonging to the *M. tuberculosis* (*Mtb)* complex supports the potential role of additional cells in the anti-mycobacterial host immune response (Figure 2). Therefore, in this section, we summarise current evidence coming from both NTM and *Mtb* complex species, although we acknowledge that there could be potential species-specific differences in cellular recruitment and response against mycobacteria.

The first group of cells proposed for further investigation is epithelial cells. Due to their anatomical location, they are among the first to encounter mycobacteria, acting as a physical, chemical, and immunological barrier [83]. However, NTM have evolved various mechanisms to promote colonization of epithelial tissue. For instance, the fibronectin attachment protein homologue (FAP-P) expressed by *M. avium* binds to host tissues through interactions with soluble and extracellular fibronectin [45]. Other adhesins, such as the antigen 85 complex (Ag85) and heparan sulphate proteoglycans (HSPGs), participate in analogous processes [46,47].

Moreover, *Mab* can disrupt epithelial barrier integrity by upregulating transcripts for certain tight junction proteins, such as claudin-1, while downregulating others, thereby facilitating bacterial translocation [84]. Additionally, *M. marinum* promotes the secretion of matrix metalloproteinase-9 (MMP-9) from surrounding epithelial cells, which induces extracellular matrix degradation [48]. These processes enhance epithelial colonization and are particularly relevant for tissue invasion.

On the other hand, evidence indicates that *Mab* and other NTM can invade multiple human epithelial cell types and replicate intracellularly [49,85,86], promoting also the induction of antimicrobial peptides such as human β-defensin-2 (HβD2) and the secretion of IL-8 via the TLR2 signalling pathway [49]. In contrast, *M. smegmatis* exhibits increased infectivity after prior passage through A549 alveolar epithelial cells, triggering an exacerbated pro-inflammatory response characterized by the secretion of IL-1β, IL-6, IL-8, TNF-α, MIP-1α, and MCP-1 [87].

Further research on the role of epithelial cells in *Mab* adherence is essential, as it represents the first step in establishing infection. It is a priority to identify the conditions under which epithelial dysregulation occurs, and how it promotes infection progression rather than bacterial clearance. Additionally, epithelial dysregulation can lead to tissue damage, potentially contributing to the development of granulomatous lesions [48].

Beyond epithelial cells, mast cells (MCs) represent an additional tissue-resident immune population implicated in anti-mycobacterial defence. Localized in skin, pulmonary, and mucosal compartments, MCs exert multifaceted functions through the release of preformed and de novo synthesized mediators, orchestrating immune cell recruitment and modulating local inflammatory responses [88].

MCs recognise NTM through pattern recognition receptors such as TLR2, which mediates activation upon interaction with *M. smegmatis* LAM, inducing cysteinyl leukotriene release [89]. MCs can also phagocytose *M. marinum*, leading to upregulation of cathelicidin, COX-2, TNF-α, and NOD2 expression [54]. However, no information is available for *Mab.*

Several studies have demonstrated that direct interaction between MCs and *M. tuberculosis* is mediated by CD48 [90], lipid rafts [91], and Toll-like receptor 2 [92]. TLR2 deficiency markedly reduces cytokine expression (IL-1β, TNF-α, IL-6, MCP-1), increases bacillary burden, and attenuates neutrophil and mononuclear infiltration in pulmonary tissue, while blockade of CD48 receptor significantly impairs histamine secretion. The overexpression of these receptors by MCs following IL-33 priming enhances the number of interactions with the bacilli [93]. Additionally, other antimicrobial mechanisms, such as the release of extracellular traps, are induced upon infection of MCs by BCG and *M. tuberculosis* [94,95].

Moreover, the role of MCs has been documented in granuloma formation. MCs localize to granulomatous, vascular, and fibrotic areas, where they exhibit a tryptase-chymase-double-positive phenotype and express IL-17. Intracellular bacilli are also observed within tryptase- and chymase-positive MC subsets [96]. Cytokines produced by MCs (IL-1β, TNF-α, MIP-1, IL-6) are associated with protective immunity and secrete mediators implicated in granuloma maintenance, including TNF-α for neutrophil activation and DC recruitment, as well as IL-12, IFN-γ, IL-6, and TGF-β for T-cell polarization [97]. Beyond granuloma formation, MCs could also be involved in *Mab* uptake via the expression of tetraspanins, which contribute to the organization of membrane proteins and the modulation of diverse signalling pathways [76].

Another cellular subset worthy of further investigation is mucosal-associated invariant T (MAIT) cells. These cells are enriched at mucosal and barrier sites such as the lungs, liver, and intestinal tract. Their activation is mediated through the interaction between their semi-invariant T-cell receptor (TCR) and the major histocompatibility complex class I-related molecule (MR1), highly conserved across different immune cells [98]. MR1 presents metabolites from the riboflavin (vitamin B2) biosynthetic pathway, which is produced by specific bacterial types, including mycobacteria [99].

Although conventional T cell responses in mycobacterial infections are well established, MAIT cells have recently emerged as critical mediators of anti-mycobacterial immunity. For instance, in active TB patients, MAIT cells travel from peripheral blood to infection sites, where they likely interact with infected APCs to promote bacterial clearance [56]. Additionally, upon in vitro exposure to *M. tuberculosis* or BCG-infected cells, MAIT cells secrete IFN-γ, a cytokine essential for bacterial containment [100,101].

On the other hand, MAIT cell depletion in murine models confers increased susceptibility to BCG and *Mab,* reflected by an increase in pathogen burden [100]. In vitro, MR1-dependent interactions between BCG-infected bone marrow-derived dendritic cells and MAIT cells promote activation, as indicated by elevated CD69 expression [101]. Finally, activated MAIT cells secrete IFN-γ, TNF-α, IL-17, and cytotoxic effector molecules such as perforin and granzyme B, factors essential for limiting mycobacterial proliferation [56].

The specificity of the cytotoxic responses against mycobacterial infection positions MAIT cells as promising targets for host-directed therapies. However, further research is required to understand which immune cells participate in these infections, their functional roles, and how they can be modulated to enhance host defence against the pathogen.

Finally, another subset of unconventional T cells is worth mentioning, namely γδ T cells. These cells comprise only 1–5% of circulating lymphocytes but are enriched in peripheral tissues such as the skin, intestines, and lungs [102]. γδ T cells recognize nonpeptidic compounds with phosphoester structures, known as phosphoantigens, which are present in several NTM, including *M. avium*, *M. smegmatis*, *M. fortuitum* and *M. marinum* [103]. In response to these antigens, γδ T cells become activated, leading to proliferation and increased production of IFN-γ [104].

Recently, accumulating evidence has highlighted the relevance of γδ T cells in the immune response against mycobacterial infections. For instance, in individuals with tuberculosis, a marked increase in the circulating frequency of this peculiar subset is observed in the blood [105]. Additionally, evidence of γδ T cell memory-like phenotype was also demonstrated when peripheral blood mononuclear cells from BCG-vaccinated individuals underwent robust clonal expansion upon stimulation with mycobacterial antigens compared to cells from non-vaccinated [106]. Notably, γδ T cell subsets from BCG-vaccinated individuals were shown to inhibit intracellular *M. avium* and *Mab* within macrophages [55]. Furthermore, in a bovine model, γδ T cell subsets were localized at the periphery of *M. avium* granuloma structures, correlating with the degree of granuloma organization [107].

The ability of γδ T cells to mount both innate-like and adaptive immune responses makes them valuable candidates for study, as they can participate at different stages of infection. However, the underlying mechanisms remain poorly understood, and further studies are required to clarify their role.

## 7. Conclusions and Perspectives

NTM, particularly *Mab*, present major research and clinical challenges due to their complex biology and ability to adapt through various mechanisms that support survival and help them evade the immune system. The pathogenesis of *Mab* involves a complex and dynamic interaction between the bacteria and the host, surface lipids, and the participation of resident and recruited immune cells.

From the *Mab* perspective, the coexistence of smooth and rough variants within the host plays a crucial role in immune evasion and persistence. This allows *Mab* to adjust its interactions with the host’s immune system, complicating the resolution of infections. It remains unclear whether the smooth and rough morphotypes evoke different specific antimicrobial immune responses, whether they target distinct immune cell populations, or whether the main difference between the two morphotypes lies in the intensity of the immune response they provoke.

While most pathomechanistic research has focused on *Mab* smooth strains, future studies should prioritize rough strains or develop models that examine infections involving both morphotypes. This approach will help clarify their contributions to chronic infections and aid in developing effective therapies for clinically relevant forms. Additionally, exploring strategies to inhibit the conversion from smooth to rough variants is vital, as this transition is associated with increased virulence and heightened inflammatory responses.

The immune response, particularly granuloma formation, is a double-edged sword from the host perspective. While it effectively contains infections, failure to eliminate the bacteria can result in chronic disease. Therefore, in addition to examining traditional immune cells, such as macrophages and lymphocytes, and exploring ways to manage their polarization towards a hyper-inflammatory state, it is essential to study non-traditional immune cells. These include epithelial, mast, MAIT, and γδ T cells, which could play a critical role in the early, localized defence against *Mab*. However, said cells may also significantly contribute to granuloma formation and persistence by influencing the surrounding inflammatory environment. Consequently, these non-classical immune subsets become interesting targets for future research.

Beyond developing novel strategies to target mycobacteria with more effective antibiotic combinations than currently available, novel approaches might include host-directed interventions aimed at targeting the hyperinflammatory cellular immune response driving granuloma formation and tissue damage.

For instance, CD38, an activation marker expressed by lymphoid and myeloid cells [115], emerges as a promising target for investigation. A deficiency of this receptor in a mouse model increases susceptibility to *M. avium* infection by impairing Th1 responses, which is considered protective [116]. Building on these findings, a clinical trial tested daratumumab, an anti-CD38 monoclonal antibody, in a patient with disseminated NTM unresponsive to IFN-γ therapy, resulting in marked clinical improvement [117]. Therefore, while CD38 is not ultimately a receptor *Mab*/NTM directly bind to, these results highlight the potential of host-directed strategies to control disease progression by quenching hyper-inflammatory responses.

In this review, we highlighted the potential roles of various immune receptors, including TLRs, complement, and other non-canonical receptors, in coordinating inflammation and immune responses to different NTM. These receptors may play a crucial role in controlling different stages of *Mab* infection. However, it is still unclear which receptors act in specific phases of the infection, or whether a hierarchy exists in the contribution of these receptors to mount an efficient antimicrobial response. Developing or repurposing targeted receptor-specific treatments, like monoclonal antibodies targeting CD18 (e.g., erlizumab, rovelizumab) [118], CD11b (ASD141) [119], CD48 (SGN-CD48A) [120] and recombinant molecules cleaving CD46 (Ad35K++) [121], could help preventing *Mab* entry, enhancing local immunity, helping in the successful eradication of *Mab* infection and preventing further tissue damage. However, more research focused on *Mab* and the potential therapeutic benefit of targeting said receptors at early or late stages of infection is needed before any conclusions can be drawn.

While experimental models and new therapeutic targets are continuously being developed, the rarity of *Mab* infections poses a significant challenge to applied clinical research. The retrospective nature of available epidemiology studies, disease registries (especially in CF), meta-analyses, single-centre cohorts, and case reports severely limits our understanding of the transmission, common facilitating host factors, and pathogenetic mechanisms involved.

Additionally, this is further complicated by patient-specific variables, as the type and intensity of the host’s immune response can vary dramatically depending on the immune status, genetic background, or the presence of comorbidities. In clinical settings, *Mab* is rarely found in isolation, as it often coexists with other NTM [122]. Although the synergistic interactions between these species remain poorly understood, such coinfections are commonly observed in immunocompromised patients. Furthermore, coinfections are not limited to NTM species [123], potentially complicating diagnosis and altering the immune responses.

The inability to perform large randomized controlled trials further slows the development and testing of novel treatment solutions. Therefore, a concerted global effort to organize multicentric, prospective studies is essential to elucidate the full scope of *Mab* infection, from pathogenesis to treatment.

Characterizing patient-specific immune heterogeneity will be critical for designing personalized interventions that combine conventional antimicrobial therapies with approaches tailored to the patient’s unique immune profile, particularly for those with chronic, co-morbid, refractory conditions, or those who are immunocompromised.

In conclusion, future efforts should focus on developing experimental and clinical models that effectively capture the complexity of *Mab* infections, allowing the evaluation of combination therapies that target both bacterial and immune modulation, to increase our understanding of such complex mycobacteria–host dynamics, evaluating the efficacy of combination therapies and improving the lives of people affected.

## Data Availability

No new data were created or analyzed in this study.
